# Hydrochar did not reduce rice paddy NH_3_ volatilization compared to pyrochar in a soil column experiment

**DOI:** 10.1038/s41598-020-76213-z

**Published:** 2020-11-05

**Authors:** Xiaoyu Liu, Yueqin Cheng, Yang Liu, Danyan Chen, Yin Chen, Yueman Wang

**Affiliations:** 1 Jiangsu Vocational College of Agriculture and Forestry, Jurong, 212400 China; 2 Institute of Agricultural Information, Jiangsu Academy of Agricultural Sciences, Nanjing, 210014 China; 3 Key Laboratory of Agro-Environment in Downstream of Yangtze Plain, Ministry of Agriculture and Rural Affairs/Institute of Agricultural Resources and Environment, Jiangsu Academy of Agricultural Sciences, Nanjing, 210014 China; 4 Nanjing Station of Quality Protection in Cultivated Land, Nanjing, 210036 China; 5 College of Horticulture, Jinling Institute of Technology, Nanjing, 211169 China

**Keywords:** Agroecology, Ecosystem ecology

## Abstract

Pyrochar (PC) is always with high pH value, and improper application might increase rice paddy ammonia volatilization (PAV), which is the main nitrogen loss through air during rice production. Differently, hydrochar (HC) takes the advantages of high productive rate and always with lower pH value compared with PC. However, effect pattern and mechanism of HC on PAV are still unclear. In the present study, soil column experiments were conducted to investigate the effect of PC and HC application on PAV. In total, treatments with four types of biochar (WPC, SPC, WHC and SHC, i.e., PC and HC prepared with wheat straw and sawdust, respectively) and two application rates (0.5% and 1.5%, w/w) were set up and non-biochar application was used as control. Results showed that, application of HC with low pH value could not reduce PAV compared with PC. Total PAV increased significantly as the increase of HC application rate (especially for WHC). The increment of PAV under high rate HC application might be due to the strong buffer capacity of soil, the aging of biochar, the high nitrogen from HC. The results indicated that HC should be pretreatment before utilization in agricultural environment considering PAV reduction.

## Introduction

Ammonia (NH_3_) volatilization emission is a key incentive of air (fog and haze) and water pollution (eutrophication)^1,2^. Paddy ammonia volatilization (PAV) is the main nitrogen (N) losses through air, and occupies 10–40% of the total nitrogen (N) fertilizer^3^. Total NH_3_ emissions from global fertilizer use were estimated at approximately 11 Tg N year^−1^^4^, and almost 1.7 Tg N year^−1^ from paddy fields in China^5^. Therefore, management to reduce PAV is of great importance for both environment and agriculture.

Pyrolysis biochar (PC, pyrochar) has been intensive studied in carbon sequestration, soil improvement and greenhouse gas mitigation^6,7^. However, PC always has a high pH value, usually ranged from 8–10, depends on different conditions^8^. Application of PC with high pH value could increase PAV by 19% on average^9,10^, especially under high rate PC application^11,12^. Hence, to reduce PAV, PC should be applied with appropriate rates^13^ or combined with other material such as wood vinegar^14^, and new biochar preparation methods should also be proposed.

Compared with PC by pyrolysis, hydrochar (HC) is produced by hydrothermal carbonization (HTC)^15^. During HTC, biomass is heated in the presence of water and autogenous pressure in an oxygen free environment^16^. HTC takes the advantages of higher char yield, lower amounts of energy during production^17,18^. Furthermore, HC is always with low pH value (3.5–6.5) and good absorption ability^19,20^, which showed a great potential to manage PAV^21^.

Most previous researches on HC are focus on the analysis in laboratory, such as comparison of physical and chemical properties of different types of HC^22,23^. Researches on HC application in agriculture environment are limited^24,25^. Considering the promising prospect for HTC technology in biomass conversion and utilization, the application of HC in agro-environment should be investigated before a comprehensive evaluation. A set of pot trials with barley, phaseolus bean, leek was conducted to determine the effects of HC on plant N availability and biomass production^26^, but without the observation of NH_3_ volatilization emission. Studies have been conducted to investigate the effects of HC on PAV, but the results were inconsistent for stimulating or inhibiting PAV, depending on the feedstock and preparation of HC^27,28^. By now, the pattern and mechanism of HC on PAV has seldom been reported, and whether HC could be an alternative of PC in consideration of PAV reduction are still unclear. Thus, soil column experiments were conducted to investigate the effect of PC and HC application on PAV in the present study. The purpose of this study is to (1) investigate and compare the effect of PC and HC on PAV; (2) investigate the correlation between properties of flood water, surface soil and PAV to understand the intrinsic mechanisms; and (3) evaluate the effects of biochar production method, feedstock and application rate to provide a reasonable biochar application scheme to manage PAV. The present study would offer a comprehensive evaluation on HC application in rice production for management of PAV.

## Methods

### Preparation and the basic properties of pyrochar and hydrochar

In the present study, two types of PC were generated from wheat straw and sawdust in an oxygen-limited pyrolysis system at 700 °C (named WPC and SPC, respectively). In detail, the temperature of the reactor was set at 700 ℃ for 8 h first. After the pyrolysis process completed, the temperature cooled to room temperature^13^. Two types of HC were also derived from wheat straw and sawdust (named WHC and SHC, respectively). Feedstock and water were mixed at a ratio of 100 g/L. The reactor was heated to 260 °C for 1 h, then cooled until reach the room temperature. The remained products were dried and passed through a 2 mm sieve. The basic properties of these 4 biochar were showed in Table 1.

**Table 1:** Basic properties of pyrochar and hydrochar used in the present study.

Type	SSA/m^2^ g^−1^	TN/g kg^−1^	pH
WPC	32.03	8.11	9.80
SPC	20.73	4.23	8.58
WHC	0.30	16.10	4.69
SHC	1.44	2.97	3.68

WPC, SPC, WHC and SHC represent pyrochar and hydrochar prepared with wheat straw and sawdust, respectively; SSA represent specific surface area; TN represents total nitrogen.

To investigate the dissolved matter from biochar in the present study, 4 types of biochar (WPC, SPC, WHC and SHC) were dipped in cold (20 °C) and hot (95 °C) deionized water for 1 h at 1:20 char/water ratio (*w/v*), respectively. The mixture was stirred for 1 h at a speed of 600 r/min. Then, the extracted water was obtained after filtration by a 0.45 μm Millipore filter. TN and COD (Chemical oxygen demand) of extracted water were measured by a San++ Continuous Flow Analyzer (Skalar, Netherlands) and a COD analyser (DR1010 COD, HACH, USA), respectively. To simulate the aging process of hydrochar, WHC and SHC were dipped in 30% v/v H_2_O_2_ for 3 h at 1:10 char/water ratio (w/v). The remained material was obtained through filtration, drying and sieving as WHC-aged and SHC-aged, respectively. The pH value of these two aged hydrochar were measured in deionized water at a ratio of 1:10 *w/v* using combined reference electrodes and a Ф255 pH/temp/mV meter (Coulter Bechman Co., USA).

### Soil column experiment design

Soils used for the column experiment was obtained from the surface layer (approximate to 20 cm) of a rice paddy (stagnic anthrosols) in Zhoutie Town, Jiangsu Province (31.4765° N, 119.9861° E). Sand, silt and clay contents were 8, 24 and 68%, respectively. The soil was with a pH value at 6.38, total nitrogen at 1.56 g kg^−1^, organic carbon at 2.28%^11^. Soil columns in the experiment were designed by polyvinyl chloride with a diameter of 30 cm and height of 50 cm. Drainage hole was designed at the bottom of each column for midseason drainage.

Four types of biochar were set at rates of 0.5% and 1.5% (*w/w*), respectively. In total, 8 biochar application treatments were set up (named as WPC-0.5%, WPC-1.5%, WHC-0.5%, WHC-1.5%, SPC-0.5%, SPC-1.5%, SHC-0.5% and SHC-1.5%, respectively). Non-biochar application treatments with N fertilizer (CKU) were set up as control. 35 kg soil were mixed up with relative biochar before filled into each soil column. Each treatment was conducted with three replicates.

Rice seedlings were transplanted on July 2, 2017, with a density of six seedlings per column, and a basal fertilizer (BF) of 96 kg N ha^−1^, 90 kg P_2_O_5_ ha^−1^, and 120 kg K_2_O ha^−1^ was applied. The tiller (TF) and panicle (PF) fertilizers were applied at a rate of 96 and 48 kg N ha^−1^ on Jul. 16 and Aug. 14, respectively. During the rice growing season, all the columns shared the same irrigation treatment, which were keeping with a 3 cm water layer, except a midseason drainage from Jul. 31 to Aug. 7, 2017. The harvested was conducted on Nov. 4, 2017.

### Measurement of paddy NH_3_ volatilization

Continuous air-flow enclosure method was introduced for measurement of PAV fluxes by using a transparent Plexiglas chamber (with 15 cm inner diameter and 20 cm height) (Feng et al. 2016). The NH_3_ absorbent contained 80 mL boric acid (2%), and mixed with methyl red, bromocresol green, and ethanol as indicator. The absorbent solution was titrated against with 0.01 M H_2_SO_4_ after taken to laboratory. PAV measurement was conducted daily from 8:00 to 10:00 a.m. for 7 days after every fertilizer applied (BF, TF and PF, respectively). The cumulative PAV was calculated by daily emission summation over the relative measuring period.

### Measurement of pH value, total nitrogen and NH4-N concentration for flood water and surface soil

Along with the PAV observation, the pH value and NH_4_-N concentration of flood water (accompanied with PAV observation) and surface soil (after PAV observation) was also measured. Flood water was sampled on 10:00 a.m. daily until the PAV measurement ended. Soils of each column were sampled after 7 days of each fertilizer application.

The pH value of flood water and soil were measured directly or in deionized water at a ratio of 1: 2.5 *w/v* using combined reference electrodes and a *Ф*255 pH/temp/mV meter (Coulter Bechman Co., USA), respectively. The TN and NH_4_-N concentration of surface flood water samples were measured by a San^++^ Continuous Flow Analyzer (Skalar, Netherlands), where those of surface soil samples were measured by sulfuric acid catalyzed digestion and boric acid titration and the similar method as water samples after extracted by 2.0 M KCl first.

### Statistical analysis

Analysis of variance was conducted to determine significant differences between different treatments. Duncan's multiple range test was conducted using *P* < 0.05 as the standard for significance. Correlation analysis between properties of flood water, surface soil, biochar application rate and PAV was conducted. Statistical analysis and figures were performed by using the SPSS 19.0, R 3.4.1 and Origin 9.0.

## Results

### Paddy NH3 volatilization fluxes pattern after fertilizer application

**Figure 1:**
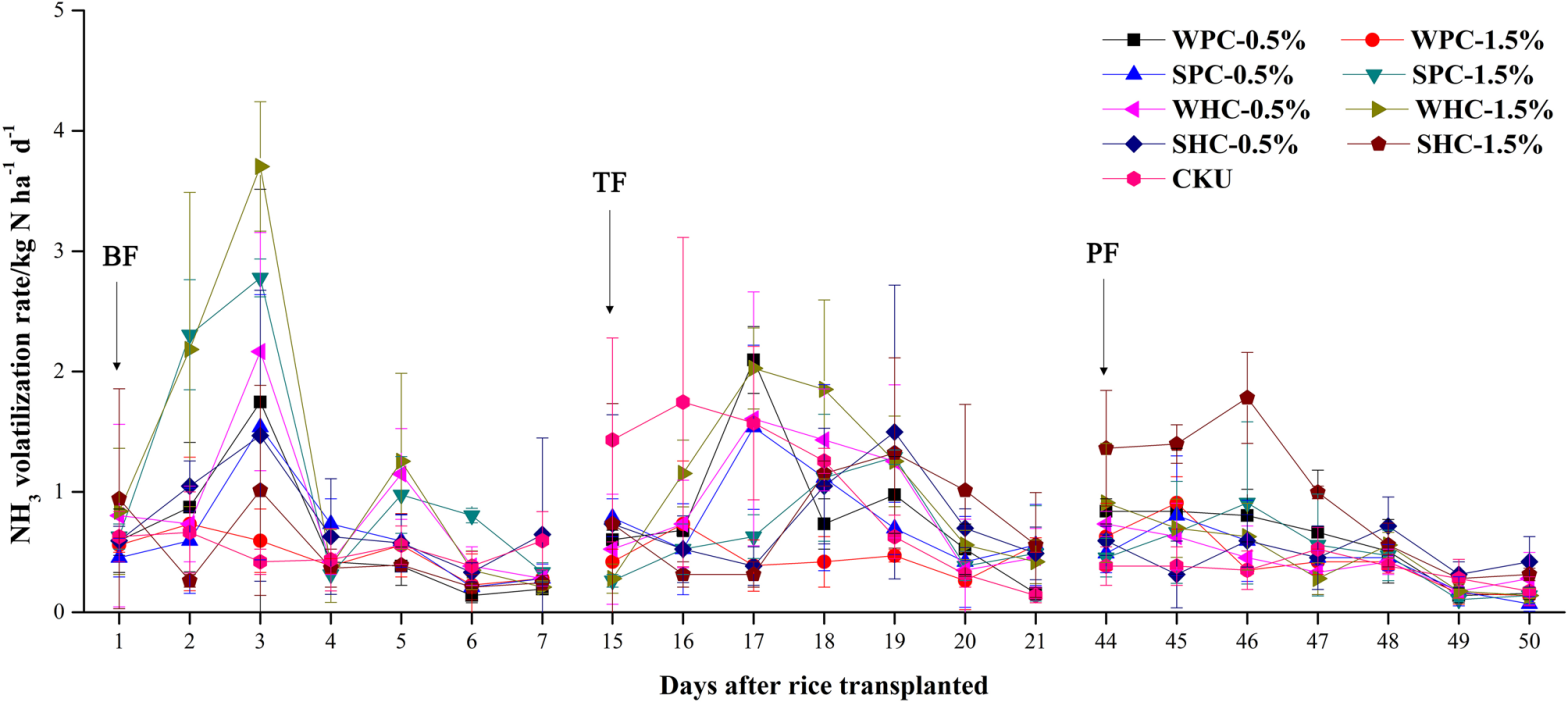
Paddy NH_3_ Volatilization fluxes of the first week after basal fertilizer (BF), tiller fertilizer (TF) and panicle fertilizer (PF) application, respectively. WPC, SPC, WHC and SHC represent pyrochar and hydrochar prepared with wheat straw and sawdust, respectively; 0.5% and 1.5% represent biochar application rates; CKU represents treatment without biochar application but with N fertilizer; Error bars represent standard deviations.

Results of PAV fluxes showed that, after each fertilizer application, PAV rates of the 9 treatments all increased at the first 3 days and decreased later until a new balance was achieved. After a 7-day observation, PAV rate of all the 9 treatments were lower than 1 kg N ha^−1^ day^−1^ (Fig. 1).

Comparison showed that, PAV of SPC-1.5%, WHC-0.5%, WHC-1.5% and SHC-0.5% after BF were significantly higher than CKU, whereas other biochar application treatments showed no significant differences. After TF, only PAV of WPC-1.5% was significantly lower than CKU. While after PF, only PAV of SHC-1.5% was significantly higher than CKU (Table 2). Total PAV after 3 fertilizer application showed that, most biochar application treatment increased PAV. Only WPC-1.5% and SPC-0.5% showed a lower total PAV than CKU, though not significantly. HC application increased total PAV, and WHC-1.5% showed significantly higher total PAV than CKU.

**Table 2:** Cumulative paddy NH_3_ volatilization of the first week after basal fertilizer (BF), tiller fertilizer (TF) and panicle fertilizer (PF) application and their summation.

Treatments	Cumulative NH_3_ volatilization (kg N ha^−1^)			
	BF	TF	PF	Total
WPC-0.5%	4.35 ± 1.72c	5.77 ± 0.22ab	4.00 ± 1.27b	14.12 ± 1.52b
WPC-1.5%	3.33 ± 0.51c	3.25 ± 1.00b	3.06 ± 0.72b	9.64 ± 1.33c
SPC-0.5%	4.40 ± 1.06c	5.64 ± 1.98ab	2.97 ± 0.94b	13.01 ± 0.87bc
SPC-1.5%	8.14 ± 0.70ab	4.70 ± 0.98ab	3.35 ± 1.82b	16.19 ± 3.09b
WHC-0.5%	6.91 ± 0.68b	6.35 ± 1.31a	3.02 ± 0.94b	15.31 ± 1.41b
WHC-1.5%	8.92 ± 1.23a	7.54 ± 1.13a	3.25 ± 0.60b	19.71 ± 2.11a
SHC-0.5%	6.72 ± 1.76b	5.38 ± 1.90ab	3.39 ± 1.25b	14.06 ± 2.57b
SHC-1.5%	3.43 ± 0.54c	5.41 ± 2.26ab	6.87 ± 1.97a	15.71 ± 3.35b
CKU	3.68 ± 0.83c	7.09 ± 2.87a	2.48 ± 0.53b	13.26 ± 1.88bc

WPC, SPC, WHC and SHC represent pyrochar and hydrochar prepared with wheat straw and sawdust, respectively; 0.5% and 1.5% represent biochar application rates; CKU represents treatment without biochar application but with N fertilizer; Values with the same letters are not significantly different at the 5% significance level.

**Figure 2:**
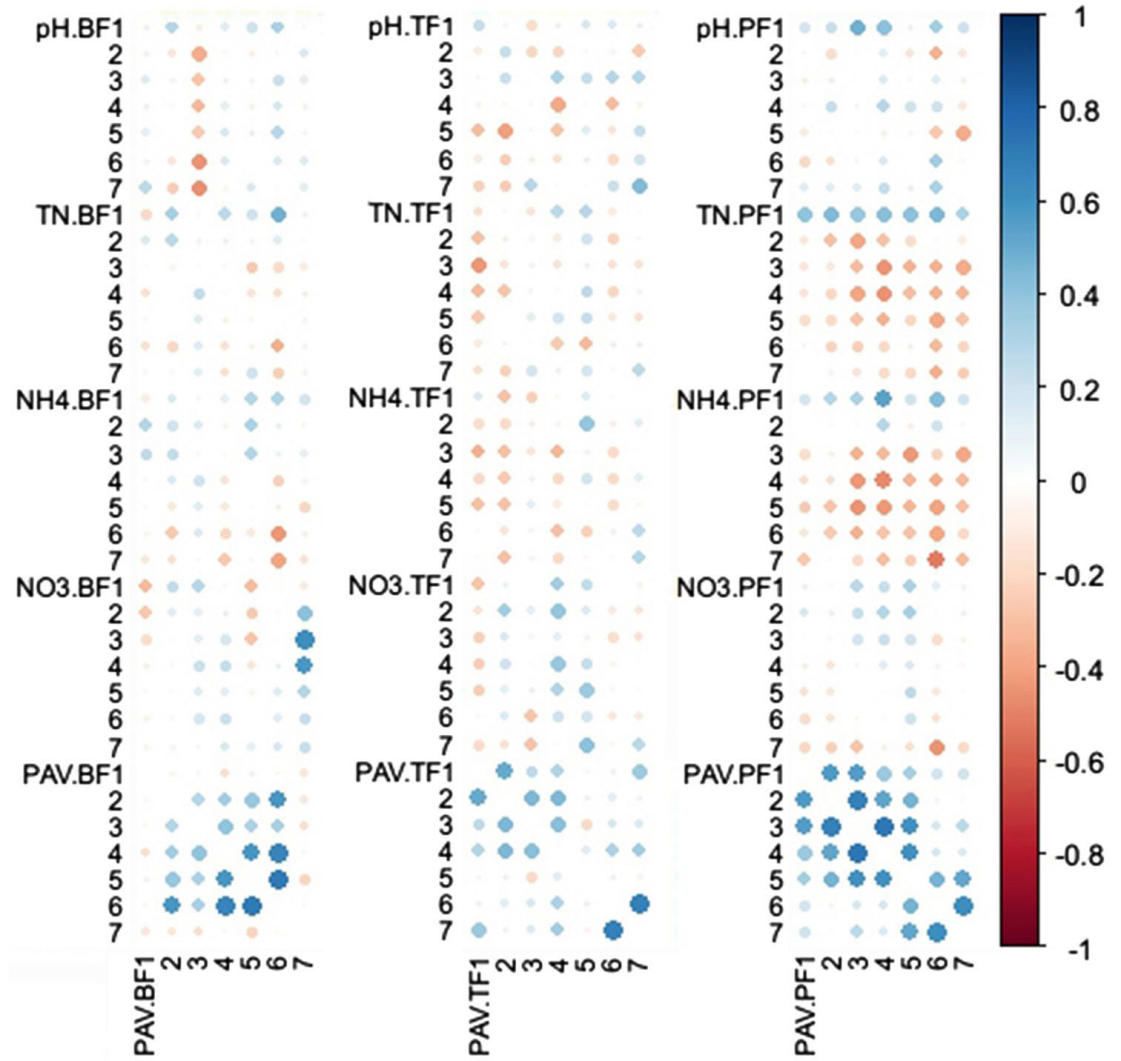
Correlation between chemical properties of flood water and PAV during the first week after basal fertilizer, tiller fertilizer and panicle fertilizer application. pH, TN, NH_4_, and PAV represent pH value, total nitrogen, NH_4_-N, and paddy NH_3_ volatilization of flood water, respectively; BF, TF and PF represent basal fertilizer, tiller fertilizer and panicle fertilizer application; number 1–7 represent the day after each fertilizer application.

Results of correlation analysis indicated that, chemical properties of flood water, e.g. pH value, TN, and NH_4_-N did not affect PAV significantly during the first week after three fertilizer application (Fig. 2). Most correlation coefficients between chemical properties of flood water and PAV in the same day (e.g. pH.BF1 and PAV.BF1) and different days (e.g. pH.BF1 and PAV.BF2) were not significant (Fig. 2). These results indicated that single daily properties of flood water could not predict daily PAV. However, daily PAV after fertilizer application were autocorrelated (e.g. PAV.BF5 and PAV.BF6). And the correlation was enhanced as the time of fertilizer application (BF ≈ TF < PF). After PF application, PAV of each single day was correlated with that next day.

### The effect of flood water on paddy NH_3_ volatilization

Properties of surface soil also showed limited impact on PAV, which was similar to that of flood water. PAV after BF and TF did not significantly correlate with its corresponding soil pH value, TN, and NH_4_-N (Table 2). In contrast, after PF, PAV were significantly correlated with soil pH value, TN and NH_4_-N. The result indicated that PAV after PF was more sensitive to surface soil properties than that after BF and TF.

### The effect of biochar application rate on paddy NH3 volatilization

In general, biochar application could increase total PAV though not significantly (r = 0.29) (Table 3). When divided by biochar prepared method (PC and HC), results showed that HC was more likely to increase PAV as the increase of application rate compared with PC. This result was in accordance with total PAV observation (Table 2). HC application rate significantly increased PAV after PF and the total PAV (r = 0.61 and 0.52, respectively). However, PC application rate showed a weak correlation with PAV (r = 0.42 at most).

**Table 3:** Correlation efficient (r) between biochar application rates and paddy NH_3_ volatilization after three fertilizer application (BF, TF and PF, respectively).

	n	PAV-BF	PAV-TF	PAV-PF	PAV-Total
Total	n = 27	0.37	− 0.05	0.34	0.29
PC	n = 15	0.42	− 0.20	0.10	0.16
HC	n = 15	0.45	0.14	0.61*	0.52*
WPC	n = 9	− 0.21	− 0.27	0.06	− 0.26
WHC	n = 9	0.88**	0.16	0.44	0.80*
SPC	n = 9	0.76*	− 0.10	0.33	0.52
SHC	n = 9	0.37	0.03	0.92**	0.51

n represents number of observations for correlation analysis; * and ** represent P < 0.05 and 0.01, respectively; WPC, SPC, WHC and SHC represent pyrochar (PC) and hydrochar (HC) prepared with wheat straw and sawdust, respectively.

Furthermore, when feedstock of biochar was considered, WPC application rate showed no significant relationship with PAV after fertilizer application (r = − 0.26 to 0.06), whereas application rate of WHC, SPC and SHC all increased total PAV after fertilizer application (r = 0.51–0.80). By comparison, WHC application rate showed a significantly impact on PAV compared with other biochar.

## Discussion

### Relationship between pH value and paddy ammonia volatilization

Soil pH value is always considered as a key factor impacts NH_3_ volatilization, and a high soil pH value could increase the risk of N loss through NH_3_ volatilization^4^. Previous researches showed that PC (with high pH value) application caused higher PAV, but if at an appropriate application rate, the PAV increment could be limited^13^. HC always had a lower pH value compared with PC^29^, and HC application was assumed to have lower PAV. However, the results of the present study showed that PAV under HC application were higher than that of PC (Table 2).

Soil and water interface are very complex. When HC and PC applied to paddy soil, the strong buffer capacity of soil could exceed their impact on pH value of both flood water and surface soil. Moreover, soil microbe might decompose the acidic organic molecule^30^, which induced the pH value of HC increase to soil pH value. The result indicated that application of biochar with low pH value, might not show a reduction of PAV. Variation of PAV emission flux induced by high rate HC application should be more related on other reasons rather than its impact on pH value.

### Relationship between nitrogen and paddy ammonia volatilization

Nitrogen of flood water and soil are the sources of NH_3_ and further affect PAV emission. Former researches showed that PAV was positively correlated with NH_4_-N of flood water^31^. However, this correlation was not observed in the present study (Fig. 2). N (TN and NH_4_-N) of flood water and soil after BF and TF showed no significant relationship with its corresponding PAV (Fig. 2 and Table 4). Only after PF, TN and NH_4_-N of soil were significantly correlated with PAV (Table 4).

**Table 4:** Correlation efficient (r) between surface soil properties and paddy NH_3_ volatilization after basal fertilizer (BF), tiller fertilizer (TF) and panicle fertilizer (PF) application.

	S-pH-BF	S-pH-TF	S-pH-PF
PAV-BF	0.13		
PAV-TF		− 0.12	
PAV-PF			0.45*

	S-TN-BF	S-TN-TF	S-TN-PF
PAV-BF	− 0.06		
PAV-TF		0.14	
PAV-PF			0.42*

	S-NH_4_-BF	S-NH_4_-TF	S-NH_4_-PF
PAV-BF	0.17		
PAV-TF		0.03	
PAV-PF			0.64**

S-pH, S-TN and S-NH_4_ represent pH value, total nitrogen and NH_4_-N of soil; * and ** represent P < 0.05 and 0.01, respectively.

The weak relationship between N (both flood water and surface soil) and PAV was mainly due to the complex interaction between soil and water. The intensive observation (each day during the first week) of the present study could not elucidate the intrinsic mechanism of biochar application on PAV. Researches showed that the inhibited activities of the NH_3_-oxidizers reduced the exhaustion of AOA on NH_3_ as a substrate, which possibly resulted in nitrification inhibition and an increase in soil NH_4_^+^-N retention^32^. For further study, soil microbial community should also be considered^33,34^.

**Figure 3:**
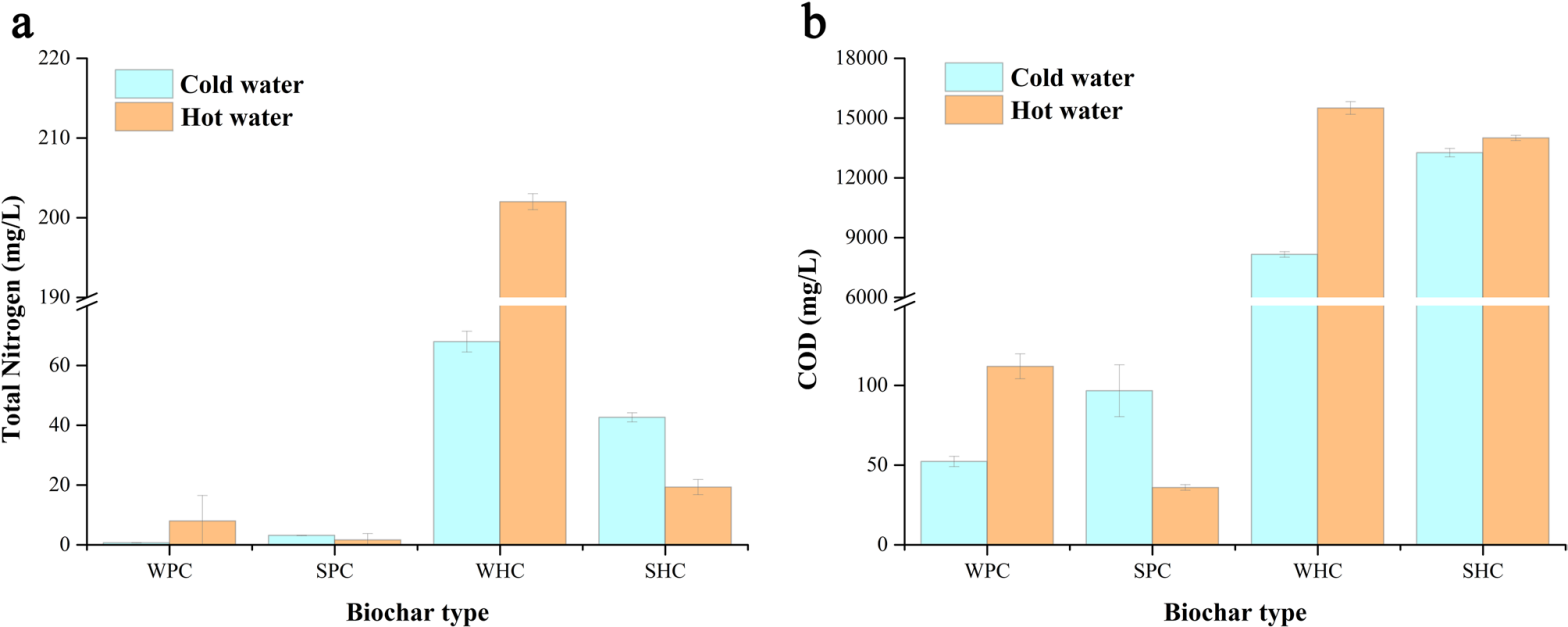
(**a**) Total nitrogen and (**b**) COD from PC and HC extracted by cold and hot water. WPC, SPC, WHC and SHC represent pyrochar and hydrochar prepared with wheat straw and sawdust, respectively; Error bars represent standard deviations.

The significant increase of PAV under WHC-1.5% treatment was mainly due to the extra nitrogen introduced from HC. The extracted water from HC had much more nitrogen and COD compared with that from PC (Fig. 3). Feedstocks showed limited effect on COD, but significant effect on TN. Wheat straw-based biochar (both WHC and WPC) showed a significantly higher TN than saw dust-based biochar. This might be due to the difference on the ratio of lignin and cellulose for different feedstocks^35^.

### Why low pH hydrochar didn’t reduce PAV?

Biochar application had multiple effects to soil, and further to PAV. Most former researches showed that PC could increase PAV due to the high pH value and extra N input^9^. However, results from some researches also indicated PC could decrease PAV due to the high specific surface area and C/N ratio^36,37^. In the present study, PC application did not show a significant impact on PAV, mainly due to its contrary effects (adsorption effect and liming effect)^9^. HC was a new type of biochar and had considerable differences in properties compared with PC. Due to the low pH value, we assumed that PAV under HC application treatment would be lower than that under PC. However, the column experiment did not prove the hypothesis, mainly as a result of strong soil buffer capacity and biochar aging.

**Figure 4:**
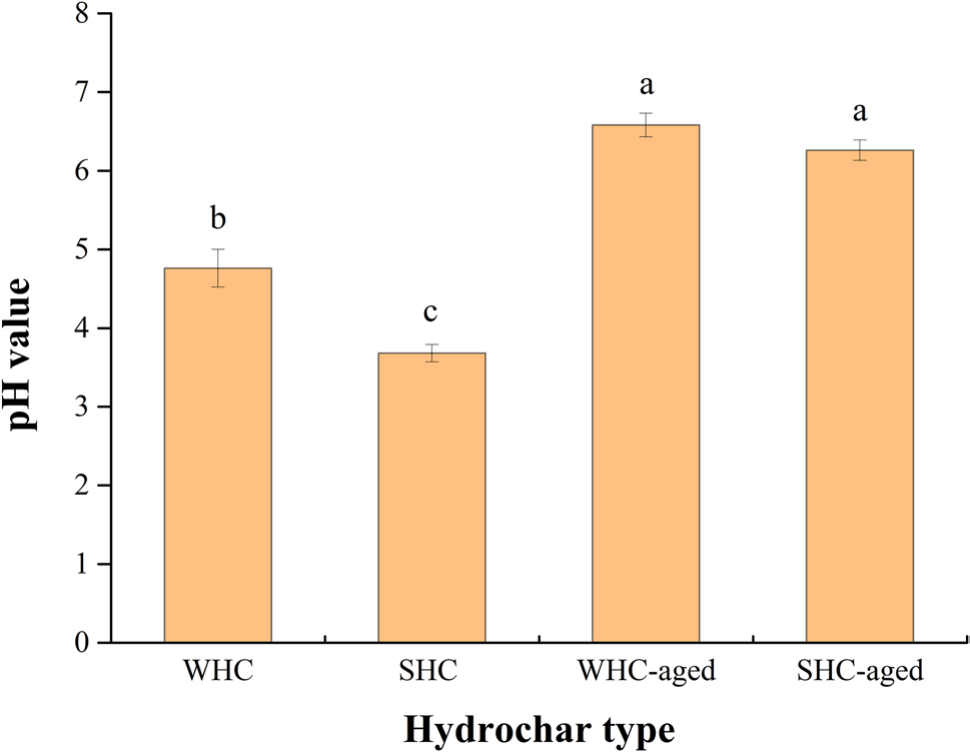
pH value of fresh and aged hydrochar. WHC and SHC represent hydrochar prepared with wheat straw and sawdust, respectively; values with the same letters are not significantly different at the 5% significance level; error bars represent standard deviations.

Though biochar was stable and could exist for centuries in soil^38^, aging of biochar should be considered for practical biochar application^39^. By simulating the aging process of hydrochar through H_2_O_2_ oxidation, the pH value of WHC and SHC turned to neutral from acidic, which was close to soil pH value (Fig. 4). In the present study, relationship between PAV and soil properties were different among three fertilizer application. After PF, pH value, TN and NH_4_-N showed significant relationship with PAV, but the same result was not observed after BF and TF. When applied to soil, biochar kept its function, e.g. high specific surface area and C/N ratio, at first. However, as the process of aging, the functionality of biochar was modified^40,41^. Therefore, significant relationship between PAV and soil properties were shown after PF. The effect of biochar aging on PAV also needed further study.

The higher PAV under high rate HC application might be due to several reasons. First, HC introduced more easily decomposed N^42^. Second, HC could release organic acid and phenol, which showed a negative effect on rice growth and its N uptake^30^. Moreover, HC application might have an impact on soil microbial community^32, 43^. Soil microbial community should be considered for further biochar application study focus on PAV. Thus, when considering PAV, biochar application should be more careful, and properties and rate of biochar should also be evaluated before application.

## Conclusion

Application of hydrochar with low pH value to rice paddy did not show a reduction on PAV compared with high pH value pyrochar. As the increase of hydrochar application rate (especially for wheat straw based hydrochar), total PAV increased significantly. Intensive measurement of soil–water properties showed a weak correlation with PAV, which indicated that mechanism of PAV should be further investigated. The increment of PAV under high rate hydrochar application might be due to the aging process and the nitrogen introduced from hydrochar. For PAV reduction, pretreatment is needed before practical application of hydrochar.
