# Author Correction: Hydrochar did not reduce rice paddy NH_3_ volatilization compared to pyrochar in a soil column experiment

**DOI:** 10.1038/s41598-021-88214-7

**Published:** 2021-04-13

**Authors:** Xiaoyu Liu, Yueqin Cheng, Yang Liu, Danyan Chen, Yin Chen, Yueman Wang

**Affiliations:** 1 Jiangsu Vocational College of Agriculture and Forestry, Jurong, 212400 China; 2 Institute of Agricultural Information, Jiangsu Academy of Agricultural Sciences, Nanjing, 210014 China; 3 Key Laboratory of Agro-Environment in Downstream of Yangtze Plain, Ministry of Agriculture and Rural Affairs/Institute of Agricultural Resources and Environment, Jiangsu Academy of Agricultural Sciences, Nanjing, 210014 China; 4 Nanjing Station of Quality Protection in Cultivated Land, Nanjing, 210036 China; 5 College of Horticulture, Jinling Institute of Technology, Nanjing, 211169 China

Correction to: *Scientific Reports* 10.1038/s41598-020-76213-z, published online 05 November 2020

The original version of this Article contained errors in the Acknowledgement.

“This work was supported by the Natural Science Foundation of China (31800358), the Jiangsu Agricultural Science and Technology Innovation Fund (CX(19)3099), the Research Fund of Key Laboratory of Agro-Environment in Downstream of Yangtze Plain (AE2018006), the Key Project of Jiangsu Science and Technology Plan (BE2019378), and the Foundation of Jiangsu Vocational College of Agriculture and Forestry (2019kj014). The authors highly appreciated the contribution of Dr. Yanfang Feng from Jiangsu Academy of Agricultural Sciences to the manuscript.”

should read:

“This work was supported by the Foundation of Jiangsu Vocational College of Agriculture and Forestry (2019kj014), the Jiangsu Agricultural Science and Technology Innovation Fund (CX(19)3099), the Natural Science Foundation of China (31800358), the Research Fund of Key Laboratory of Agro Environment in Downstream of Yangtze Plain (AE2018006), and the Key Project of Jiangsu Science and Technology Plan (BE2019378). The authors highly appreciated the contribution of Dr. Yanfang Feng from Jiangsu Academy of Agricultural Sciences to the manuscript."

